# Acute Glucose Shift Induces the Activation of the NLRP3 Inflammasome in THP-1 Cells

**DOI:** 10.3390/ijms22189952

**Published:** 2021-09-15

**Authors:** Ji Yeon Lee, Yup Kang, Hae Jin Kim, Dae Jung Kim, Kwan Woo Lee, Seung Jin Han

**Affiliations:** 1 Department of Endocrinology & Metabolism, Ajou University School of Medicine, 164 World Cup-ro, Yeongtong-gu, Suwon 16499, Korea; labcelltherapy@gmail.com (J.Y.L.); jinkim@ajou.ac.kr (H.J.K.); djkim@ajou.ac.kr (D.J.K.); LKW65@ajou.ac.kr (K.W.L.); 2 Department of Physiology, Ajou University School of Medicine, 164 World Cup-ro, Yeongtong-gu, Suwon 16499, Korea; kangy@ajou.ac.kr

**Keywords:** glycemic variability, hypoglycemia, hyperglycemia, inflammasome, reactive oxygen species

## Abstract

We aimed to investigate the effect of acute glucose shift on the activation of the NLRP3 inflammasome, IL-1β secretion, and underlying signaling pathways in THP-1 cells. THP-1 cells were divided into four groups and exposed to the following glucose concentrations for 24 h: constant normal glucose (NG, 5.5 mM), constant high glucose (HG, 25 mM), normal to high glucose shift (NG-to-HG, 5.5 to 25 mM), and high to normal glucose shift (HG-to-NG, 25 to 5.5 mM). Cell viability, oxidative stress, and the levels of NLRP3 inflammasome components were assessed. Both directions of the acute glucose shift increased the activation of the NLRP3 inflammasome, generation of reactive oxygen species (ROS), and expression of phosphorylated p38 MAPK, JNK, and NF-κB compared with either constant NG or HG. Treatment with N-acetylcysteine, a pharmacological antioxidant, inhibited the acute glucose shift-induced generation of ROS, activation of NLRP3 inflammasome, and upregulation of MAPK-NF-κB. Further analysis using inhibitors of p38 MAPK, JNK, and NF-κB indicated that acute glucose shifts promoted IL-1β secretion by activating the signaling pathway in a ROS-MAPK-NF-κB-NLRP3 inflammasome in THP-1 cells. These findings suggested that acute changes in glucose concentration might cause monocyte inflammation, which is associated with diabetic complications.

## 1. Introduction

Recent emerging evidence has indicated that both fluctuant and sustained hyperglycemia are associated with diabetic complications [1,2]. Furthermore, several epidemiological studies have demonstrated that glycemic variability is a stronger predictor for microvascular and macrovascular complications than sustained hyperglycemia in patients with diabetes [3,4,5]. Even in the general population, blood glycemic variability has been associated with future cardiovascular events and all-cause mortality [6]. 

Despite evidence from epidemiological studies demonstrating the crucial role of glycemic variability in disease prognosis, the mechanism by which glycemic variability aggravates disease progression has not yet been fully understood. In patients with type 2 diabetes, glycemic variability triggers increased oxidative stress and endothelial dysfunction compared with chronic sustained hyperglycemia [7,8,9]. Previous experimental studies reported that glycemic variability enhanced the levels of reactive oxygen species (ROS), the proportion of inflammatory cytokines in endothelial cells, as well as the adhesion of monocytes to endothelia cells compared with stable hyperglycemia, all of which are known mechanisms linked with vascular injury [10,11,12]. Despite the knowledge that the overproduction of ROS and inflammatory cytokines caused by glycemic variability contributes to tissue dysfunction, the effect of glycemic variability on innate immune response remains unknown.

Monocytes and macrophages are central cells of the innate immune system that mediate the production of various proinflammatory cytokines. Interleukin (IL)-1β is a multifunctional proinflammatory cytokine known to play the role of an early mediator of the innate immune response, inducing the secretion of other proinflammatory cytokines, such as IL-6, IL-8, and tumor necrosis factor-α [13]. In particular, IL-1β is produced as an inactive 31-kDa precursor, termed pro-IL-1β. The conversion of pro-IL-1β to bioactive IL-1ß (17-kDa) is processed by caspase-1, a cysteine protease regulated by the activation of the inflammasome [14]. The inflammasome is a cytosolic multiprotein complex involved in innate immunity that is activated in response to various pathogen- or damage-associated molecular patterns using many different nucleotide binding and oligomerization domain (NOD)-like receptors (NLRs) [14]. The NOD-like receptor family pyrin domain-containing 3 (NLRP3) is the most characterized inflammasome. Briefly, NLRP3 triggered by stimuli is known to form a complex with its adaptor, apoptosis-associated speck-like domain containing a caspase-recruitment domain (ASC), and procaspase-1, resulting in the activation of caspase-1, which cleaves the inactive precursor pro-IL-1β and pro- IL-18 into its bioactive form. 

In particular, the innate immune system-regulated abnormal activation of the NLRP3 inflammasome has been associated with the onset of diabetes, the progression and complications of the disease, and the pathogenesis of various inflammatory diseases [15,16,17]. Mice deficient in inflammasome components are protected from high-fat diet-induced obesity, hyperinsulinemia, adipocyte hypertrophy, and macrophage infiltration [18]. In addition, monocytes derived from newly identified patients with untreated type 2 diabetes displayed increased NLRP3 inflammasome activation compared with healthy subjects [19]. Furthermore, treatment with an NLRP3 inhibitor (MCC950) counteracted the increased vascular ROS generation and improved endothelial dysfunction in a diabetic model (db/db mice) [20]. These findings signify the crucial role of the inflammasome in diabetes and many metabolic diseases and suggest the inhibition of the inflammasome as a potential therapeutic strategy.

It is known that high levels of glucose might provide the first signal to promote the transcription of IL-1β mRNA and subsequent production of pro-IL-1β [21]. In addition, high levels of glucose have been reported to induce the production of ROS, which as a secondary signal could promote the activation of the NLRP3 inflammasome and secretion of bioactive IL-1β [22]. To date, some studies have reported that the NLRP3 inflammasome can be activated in response to chronic hyperglycemia [23,24,25,26]; however, little is known about the effect of acute glucose shifts on the activation of the NLRP3 inflammasome in THP-1 cells, given that THP-1 is a human leukemia monocytic cell line extensively used as a model to estimate the modulation of monocyte and macrophage activities [27]. 

Therefore, we investigated whether acute glucose shifts could induce an increased activation of the NLRP3 inflammasome and secretion of IL-1β in THP-1 cells compared with those having a steady glucose status. To mimic in vivo glycemic variability conditions, we rapidly shifted cultured cells from normal (5.5 mM) to high glucose (25 mM) (NG-to-HG) and from high to normal glucose (HG-to-NG) medium. This study also aimed to determine the signaling pathways underlying the acute glucose shift-induced activation of the NLRP3 inflammasome. 

## 2. Results

### 2.1. Acute Glucose Shift Did Not Alter the Viability and Proliferation of THP-1 Cells

**Figure 1:**
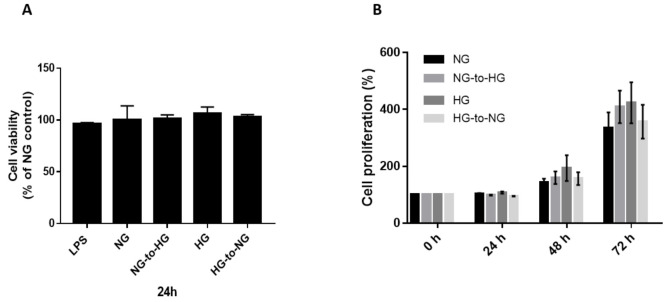
Effects of acute glucose shifts on cell viability and proliferative activity of THP-1 cells. (**A**,**B**) THP-1 cells were cultured separately in normal glucose (NG, 5.5 mM) and high glucose (HG, 25 mM). Some of them were exposed to a glucose shift (NG-to-HG or HG-to-NG). Lipopolysaccharide (LPS, 1 μg/mL) was used as a positive control. Cell viability and proliferation were assesses at the indicated incubation time points. Data are expressed as the mean ± SEM. The cell survival ratio is expressed as a percentage of the NG control.

Investigation of the effect of acute glucose shift on cell survival using the cell counting kit-8 (CCK-8) method did not reveal any significant difference in cell viability between groups (Figure 1A). Next, we examined the cell proliferation activity at 24, 48, and 72 h. We found that cell proliferation was increased in all groups over time, with no significant differences observed between groups. 

### 2.2. Acute Glucose Shift Induced the Activation of the NLRP3 Inflammasome in THP-1 Cells 

**Figure 2:**
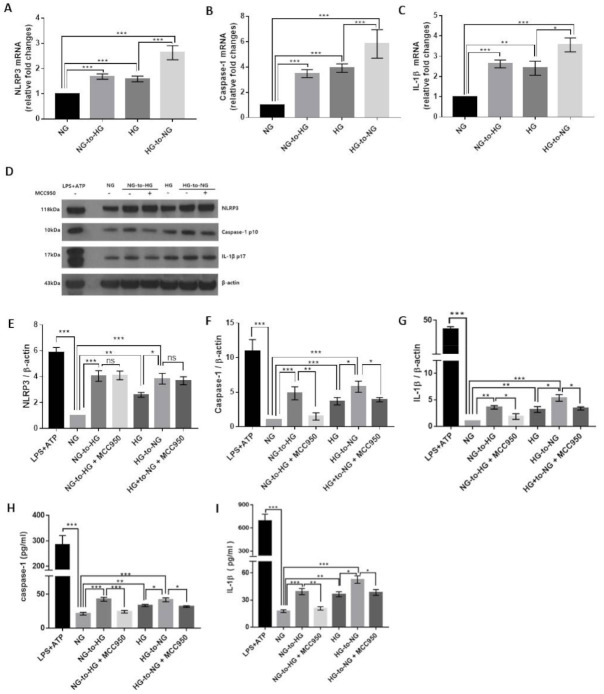
Acute glucose shift induces the activation of the NLRP3 inflammasome in THP-1 cells. The media of THP-1 cells assigned as NG- and HG-cultured cells were changed into HG and NG media, as indicated. (**A**–**C**) The mRNA levels of NLRP3, caspase-1, and IL-1β were examined by real-time quantitative PCR. (**D**) Cell lysates were subjected to western blotting using antibodies against NLRP3, caspase-1, and IL-1β. THP-1 cells were pretreated with MCC950 (10 μM) for 2 h and then exposed to acute glucose shift for 24 h. For positive control, THP-1 cells were treated with LPS (1 μg/mL) for 4 h and with 5 mM ATP for the last 30 min. (**E**–**G**) The protein expression levels of NLRP3, caspase-1, and IL-1β were quantified using Image J software. (**H**,**I**) The levels of caspase-1 and IL-1β in the culture supernatant were determined using ELISA. Data are presented as the mean ± SEM from at least three independent experiments. * *p* < 0.05, ** *p* < 0.01, *** *p* < 0.001, ns, not significant.

We examined the effect of acute glucose shifts on the transcription of components of the NLRP3 inflammasome. As shown in Figure 2A–C, the mRNA levels of NLRP3, caspase-1, and IL-1β were increased in cells exposed to an acute glucose shift compared with cells grown under constant NG or HG, respectively. Interestingly, this was true, not only for cells exposed to an NG-to-HG shift but also for those subjected to an HG-to-NG shift. However, no significant change was observed in the mRNA expression level of ASC (data not shown). 

Subsequently, we performed western blotting to analyze the protein levels of these genes in the cell lysates and culture supernatants. We accordingly found that cells exposed to an NG-to-HG shift showed increased protein levels of NLRP3, mature caspase-1 p10 and IL-1β p17 compared with cells grown under constant NG conditions (Figure 2D–G). We further observed that an HG-to-NG shift upregulated the protein components of the NLRP3 inflammasome compared with constant HG (Figure 2D–G). Both acute glucose shifts (NG-to-HG shift and HG-to-NG shift) increased caspase-1 and IL-1β secretion in the culture supernatant compared with either constant NG or HG (Figure 2H,I and Figure S1). Inhibition of the NLRP3 inflammasome by MCC950 diminished the acute glucose shift-induced increased caspase 1 and IL-1β production (Figure 2F–I). We confirmed acute glucose shift-induced NLRP3 inflammasome activation in phorbol-12-myristate 13-acetate (PMA)-induced THP-1 macrophages and primary murine macrophages (Figure S1).

We also observed that constant HG enhanced the expression of NLRP3, caspase-1, and IL-1β at both the mRNA and protein levels compared with constant NG, confirming that high glucose induced the activation of the NLRP3 inflammasome (Figure 2A–G). 

To rule out the effect of the changes in osmolality accompanying the glucose shift, mannitol was used as a hyperosmolar control (Figure S2). We accordingly found that an NG-to-HM shift (HM, 5.5 mM glucose + 19.5 mM mannitol) did not increase the expression of the components of the NLRP3 inflammasome as seen in the cells exposed to the NG-to-HG shift. 

### 2.3. Acute Glucose Shift Activated the NLRP3 Inflammasome Mediated by ROS

**Figure 3:**
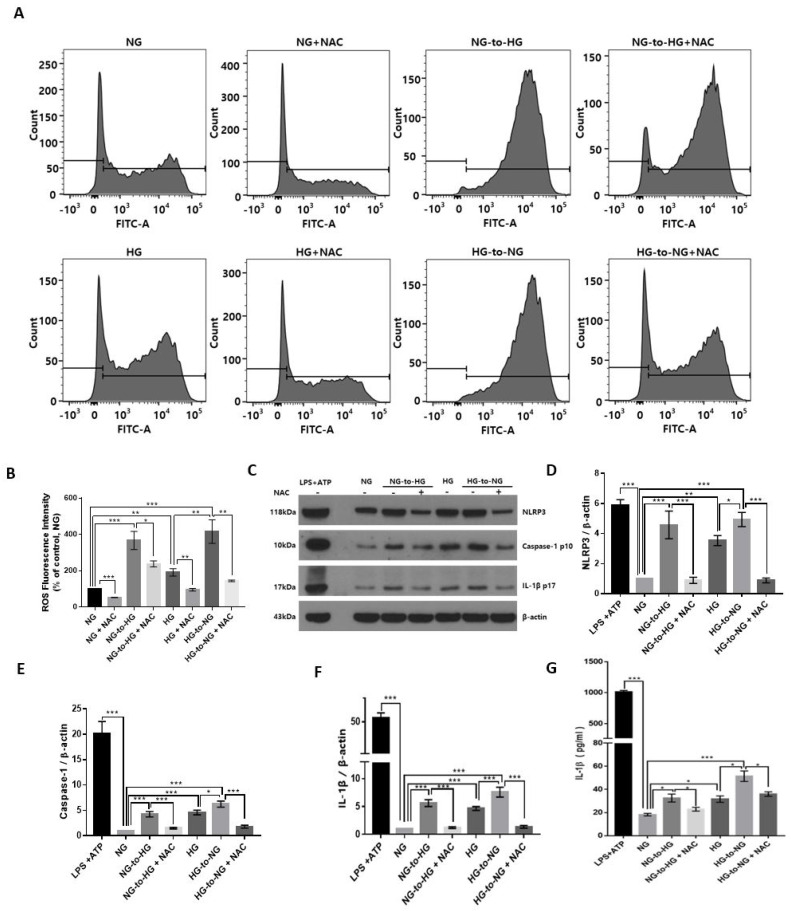
Effect of the acute glucose shift-induced generation of ROS on the activation of the NLRP3 inflammasome in THP-1 cells. Before any glucose shift, cells were pretreated with a pharmacological antioxidant, NAC (10 mM) for 1 h and then exposed to a glucose shift for 24 h. (**A**) The intracellular ROS level was measured by flow cytometry using CM-H2DCFDA. (**B**) Mean fluorescence intensity of the levels of ROS is presented as a percentage of the NG control. (**C**) Cell lysates were subjected to western blotting using antibodies against NLRP3, caspase-1, and IL-1β. (**D**–**F**) The protein expression levels of NLRP3, caspase-1, and IL-1β were quantified using ImageJ. (**G**) The level of IL-1β in the culture supernatant was determined by ELISA. Data are presented as the mean ± SEM from at least three independent experiments. * *p* < 0.05, ** *p* < 0.01, *** *p* < 0.001.

The result of FACS analysis showed that the relative level of ROS was significantly elevated under conditions of an acute glucose shift, either NG-to-HG or HG-to-NG compared with constant NG and constant HG, respectively (Figure 3A,B). To further investigate the effect of the acute glucose shift-induced levels of ROS on the activation of the NLRP3 inflammasome, we employed N-acetyl-L-cysteine (NAC) as a pharmacological antioxidant. Before exposure of cells to an acute glucose shift, cells were pretreated with NAC (10 mM) for 1 h. We observed that treatment of cells with NAC significantly inhibited the acute glucose shift-induced (both NG-to-HG and HG-to-NG) generation of ROS and expression of NLRP3 inflammasome-related proteins (NLRP3, caspase-1, and IL-1β) (Figure 3B–F). ELISA revealed that NAC also decreased the levels of the acute glucose shift-induced secretion of IL-1β (Figure 3G). 

### 2.4. Acute Glucose Shift Activated the p38 MAPK, JNK and NF-κB Signaling Pathway Mediated by ROS

**Figure 4:**
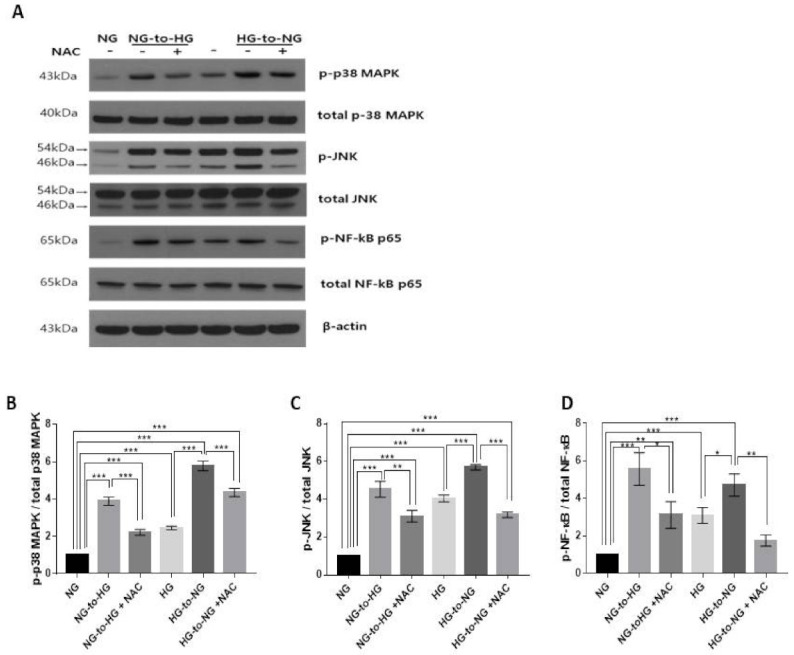
Acute glucose shift-induced ROS activates the p38 MAPK, JNK and NF-κB signaling pathways. Before exposure of cells to an acute glucose shift, cells were pretreated with NAC (10 mM) for 1 h. Thereafter, cells were incubated under conditions of a glucose shift for 24 h. (**A**) Cell lysates were subjected to western blotting using antibodies against p-p38 MAPK, p-JNK, and p-NF-κB; (**B**–**D**) The protein expression levels of p-p38 MAPK, p-JNK, and p-NF-κB were quantified using ImageJ. Data are presented as the mean ± SEM from at least three independent experiments. * *p* < 0.05, ** *p* < 0.01, *** *p* < 0.001.

To clarify the signal cascades associated with the acute glucose shift-induced generation of ROS, we assayed the expression levels of proteins of mitogen-activated protein kinase (MAPK) (p38 MAPK and Jun N-terminal kinase (JNK)) and nuclear factor-κB (NF-κB) signaling in THP-1 cells exposed to an acute glucose shift. As shown in Figure 4, the expression levels of phosphorylated p38 MAPK, JNK, and NF-κB were significantly increased after an acute glucose shift (NG-to-HG and HG-to-NG) compared with those in cells grown in constant NG or HG, respectively. However, we found that pretreatment with NAC attenuated the acute glucose shift-induced phosphorylation levels of p38 MAPK, JNK, and NF-κB. This result suggested that ROS generation was upstream of the MAPK and NF-κB pathways. 

### 2.5. Acute Glucose Shift-Induced Activation of the NLRP3 Inflammasome Was p38 MAPK, JNK/NF-κB-Dependent

**Figure 5:**
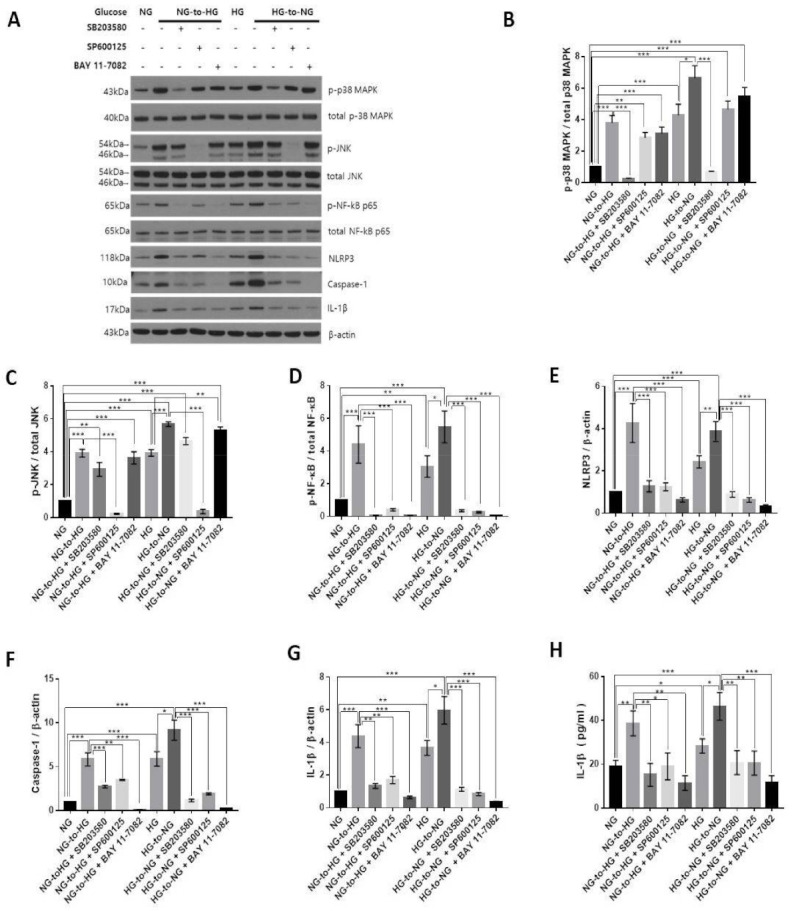
Acute glucose shift-induced activation of the NLRP3 inflammasome is p38 MAPK, JNK /NF-κB-dependent. THP-1 cells were pretreated with SB203580 (10 μM), SP600125 (20 μM), and Bay 11-7082 (10 μM) for 2 h and then exposed to an acute glucose shift for 24 h. (**A**) Western blotting was performed to detect the expression of p-p38 MAPK, p-JNK, p-NF-κB, NLRP3, caspase-1, and IL-1β. (**B**–**G**) The expression levels of p-p38 MAPK, p-JNK, p-NF-κB, NLRP3, caspase-1, and IL-1β were quantified using ImageJ. (**H**) The level of IL-1β in the culture supernatant was determined by ELISA. Data are presented as the mean ± SEM from at least three independent experiments. * *p* < 0.05, ** *p* < 0.01, *** *p* < 0.001.

To explore the involvement of MAPK and NF-κB in the acute glucose shift-induced activation of the NLRP3 inflammasome, SB203580 (inhibitor of p38 MAPK, 10 μM), SP600125 (inhibitor of JNK, 20 μM) and Bay 11-7082 (inhibitor of NF-κB, 10 μM) were used to pretreat THP-1 cells before exposure to an acute glucose shift. We accordingly found that SB203580, SP600125, and Bay 11-7082 significantly inhibited the protein expression levels of components of the NLRP3 inflammasome (NLRP3, caspase-1, and IL-1β) (Figure 5A,E–G). As shown in Figure 5I, all inhibitors markedly reduced the secretion of IL-1β compared with cells exposed to an acute glucose shift in the absence of inhibitors. In addition, we observed that the MAPK inhibitors, SB203580 and SP600125, significantly reduced the expression of NF-κB, whereas Bay 11-7082 had no effect on the expression of phosphorylated p38 MAPK and JNK (Figure 5A–D). These results indicated that in acute glucose shift-induced THP-1 cells, both p-p38 MAPK and p-JNK regulated the activation of the NF-κB and NLRP3 inflammasome, with the NF-κB pathway mediating the activation of the NLRP3 inflammasome. Combined with our aforementioned results, these data suggested that both the NG-to-HG and HG-to-NG shifts activated the NLRP3 inflammasome through a ROS-induced upregulation of the MAPK/NF-κB pathways in THP-1 cells.

## 3. Discussion

**Figure 6:**
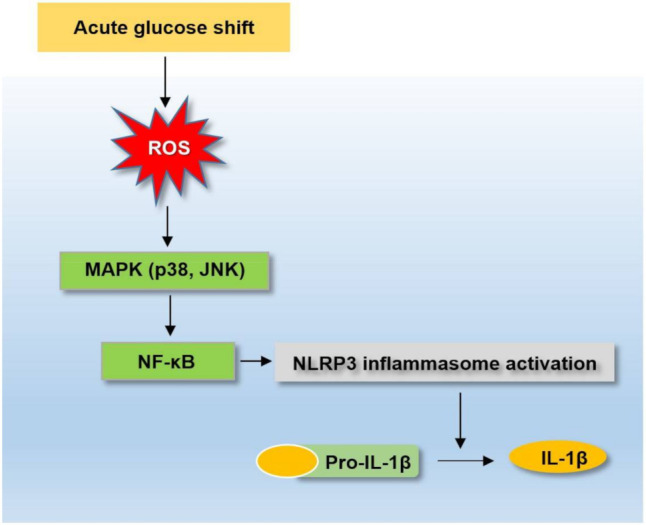
Schematic diagram depicting the acute glucose shift-induced activation of the NLRP3 inflammasome. An acute glucose shift induces the activation of the NLRP3 inflammasome, which is mediated by the activation of the MAPK and NF-κB signaling pathways through the generation of ROS.

Accumulating evidence has suggested that glycemic variability exerts more harmful effects than chronic hyperglycemia in the development of diabetic complications [7]. In addition, technical advances in devices for monitoring blood glucose levels that can measure glycemic variability in clinical practice have increased the interest of researchers into the role of glycemic variability in the progression of diabetes. To the best of our knowledge, this was the first study to demonstrate that not only an NG-to-HG but also an HG-to-NG shift could induce a greater activation of the NLRP3 inflammasome than conditions of constant NG or HG, respectively, in THP-1 cells. Our findings suggested that the acute glucose shift-induced (NG-to-HG and HG-to-NG shift) overproduction of ROS could lead to the activation of the NLRP3 inflammasome mediated by the MAPK/NF-κB signaling pathways (Figure 6). We used acute glucose shift conditions as changes between 5 mM and 25 mM glucose to emulate the commonly observed glycemic variability owing to postprandial hyperglycemia in patients with type 2 diabetes, rather than rare severe hypoglycemia. Owing to the difficulties in collecting human primary monocytes, the THP-1 monocyte-like cell line, derived from acute monocytic leukemia, has been extensively used in the study of NLRP3 inflammasome biology, despite its tumoral origin [28]. 

Emerging evidence has indicated that the activation of the NLRP3 inflammasome might lead to the maturation and secretion of IL-1β, which has been implicated in the pathological development of type 2 diabetes and cardiovascular diseases [29]. However, most studies on diabetes-related diseases and the NLRP3 inflammasome have been performed under constant hyperglycemia. To the best of our knowledge, there have been no studies on glycemic variability and the activation of the NLRP3 inflammasome. In this study, we found that an acute glucose shift activated the NLRP3 inflammasome to a greater extent than chronic NG or HG conditions in THP-1 cells. We also confirmed that an acute glucose shift induced the increased production of ROS compared with constant NG or HG conditions. Oxidative stress refers to elevated intracellular levels of ROS that can act as a second messenger, regulating inflammation responses and leading to organ damage [30]. Because the production of ROS has been identified as a key factor in the activation of the NLRP3 inflammasome, we investigated the molecular mechanism between ROS and the NLRP3 inflammasome under conditions of acute upward and downward glucose shifts.

The MAPK proteins are a family of serine/threonine kinases known to regulate a wide variety of cellular processes, such as proliferation, differentiation, apoptosis, and stress responses. Especially, MAPKs have been reported to play key regulatory roles in the production of these proinflammatory cytokines and downstream signaling events [31]. Likewise, NF-κB is known to modulate gene expression in diverse cellular processes, including the innate immune response and inflammation. It has been shown that ROS can both activate and repress NF-κB signaling depending on the duration and type of exposure [32]. Therefore, we hypothesized that the acute glucose shift-induced generation of ROS might be associated with the MAPK-NF-κB pathways, resulting in the activation of the NLRP3 inflammasome. Our result demonstrated that the phosphorylation of p38 MAPK, JNK, and NF-κB were significantly increased in THP-1 cells exposed to acute NG-to-HG and HG-to-NG glucose shifts compared with cells grown under constant NG and constant HG, respectively. After treatment with NAC, an antioxidant, the acute glucose shift-induced expression of p38 MAPK, JNK, and NF-κB and activation of the NLRP3 inflammasome were significantly suppressed. These results suggested that the acute glucose shift-induced ROS plays an essential role in the activation of the NLRP3 inflammasome, with the MAPK and NF-κB signaling pathways functioning as downstream signals of ROS generation. We also showed that both p38 MAPK and JNK inhibitors suppressed the expression of NF-κB and activation of the NLRP3 inflammasome, whereas an NF-κB inhibitor abolished the acute glucose shift-induced activation of the NLRP3 inflammasome, but it had no effect on the expression of phosphorylated p38 MAPK and JNK. Combined with the above-mentioned results, we assumed that an acute glucose shift might induce the secretion of IL-1β by activating the signaling pathway in a ROS-p38 MAPK, JNK-NF-κB-NLRP3 inflammasome sequence in THP-1 cells.

We found that MAPK is upstream of NF-κB in the crosstalk between MAPK and NF-κB signaling in acute glucose shift-induced THP-1 cells. Our findings were consistent with those of previous studies, which showed high glucose-induced MAPK-dependent NF-κB activation in osteoclasts [33], LPS-induced JNK dependent NF-κB activation in macrophages [34], and okadaic acid-induced extracellular signal-regulated kinase (ERK)1/2 and JNK dependent NF-κB activation in human monocytes [35]. However, crosstalk between MAPK and NF-κB signaling showed different results depending on the cell type and stimuli; NF-κB has been observed to be either upstream of MAPKs or parallel [36,37,38]. As for the MAPK/NF-κB and NLRP3 inflammasome crosstalk, there have been similar recent studies, which showed high glucose-induced MAPK/NF-κB-dependent NLRP3 inflammasome activation in osteoclasts and JNK-dependent NLRP3 activation in cardiomyocytes [33,39]. 

In our study, both NG-to-HG and HG-to-NG shifts demonstrated a similar effect on the production of ROS and activation of the p38 MAPK, JNK /NF-κB signaling pathways without changing the viability of THP-1 cells. ERK was found to be mainly involved in the NG-to-HG shift-induced activation of the NLRP3 inflammasome, but it had relatively less effect on the HG-to-NG shift (Figure S3). However, other studies using neural cells have shown conflicting results depending on the direction in which glucose levels rise and fall. For example, Hsieh et al. showed that in microglial cells, an acute upward change in glucose levels (from 5.5 mM to 25 mM) increased cell proliferation, ROS production, and JNK and p38 MAPK expression, but it resulted in the downregulation of pNF-κB. In contrast, a downward change in glucose levels (from 25 mM to 5.5 mM) was not associated with the generation of free radicals but led to increased p38 MAPK and NF-κB expression and cell death [40]. In another study conducted by Quincozes-Santos et al. using astroglial cells, both upward (from 6 mM to 12 mM) and downward (from 12 mM to 0 mM) glucose shifts were reported to decrease cell proliferation and increase the production of ROS and activation of p38 MAPK and NF-κB. Furthermore, these metabolic alterations were shown to be more pronounced in the downward glucose shift state, despite glucose deprivation being an extremely rare condition in clinical practice [41]. The latter study showed a pattern similar to our results. Piarulli et al. reported that monocytes from patients with type 2 diabetes and healthy subjects induced a significant increase in the levels of IL-1β only under conditions of low glucose (2.5 mM) but not under normal (5.0 mM) or high (20 mM) glucose conditions [42]. These glucose shift-depending discrepancies might be due to differences in the cell types used and experimental design adopted, such as glucose concentrations and incubation times. Therefore, further studies are needed to investigate whether an upward or downward shift in the levels of glucose might have different metabolic effects. 

In conclusion, we provided evidence that an acute glucose shift, not only NG-to-HG but also HG-to-NG, increased the maturation and secretion of IL-1β in THP-1 cells compared with those grown under steady glucose states (either NG or HG). These phenomena were demonstrated to be mediated by the ROS-dependent MAPK (p38, JNK)-NF-κB-NLRP3 inflammasome signaling pathways. These findings suggested that abrupt changes in glucose concentration might be more harmful to monocyte inflammation than constant normal or high glucose conditions. Thus, it was implied that reducing glycemic variability might be an important treatment strategy for the prevention of diabetic complications. 

## 4. Materials and Methods

### 4.1. Cell Culture and Cellular Treatments 

The THP-1 cell line was cultured in RPMI 1640 medium (Corning, Manassas, VA, USA), supplemented with 10 % fetal bovine serum (Corning, Woodland, CA, USA), 2 mM glutamine, 1 mM sodium pyruvate, 10 mM HEPES, 100 IU/mL penicillin, and 100 μg/mL streptomycin at 37 °C and 5 % CO_2_. Before performing any experiments, THP-1 cells were separately cultured in normal (NG, 5.5 mM) and high (HG, 25 mM) glucose media for 3 days. We divided THP-1 cells into four groups according to the glucose concentration of the media as follows: (1) NG (5.5 mM), (2) HG (25 mM), (3) NG-to-HG shift (cells cultured in NG medium replaced by HG medium, 5.5 mM to 25 mM), (4) HG-to-NG shift (cells cultured in HG medium replaced by NG medium, 25 mM to 5.5 mM). The media in the constant NG and HG groups were simply replaced by fresh media of the same glucose concentration. 

For inhibitor studies investigating the mechanism mediated by the acute glucose shift-induced activation of the NLRP3 inflammasome, cells were incubated with each glucose concentration mentioned above in the presence and absence of the pharmacological antioxidant, NAC (10 mM) (Sigma, St Louis, MO, USA), MCC950 (inhibitor of the NLRP3 inflammasome, 10 μM, Sigma), SP600124 (inhibitor of JNK, 20 μM), SB203580 (inhibitor of p38 MAPK, 10 μM), and BAY 11-7082 (inhibitor of NF-κB, 10 μM) (Enzo Life Sciences, Farmingdale, NY, USA). The NAC was dissolved in DMSO and filtered through a 0.22 μm syringe filter with a nylon membrane (Thermo Scientific, Rochester, NY, USA) and then diluted to a working concentration using the respective medium. SP600124, SB203580, and BAY 11-7082 were dissolved in DMSO.

### 4.2. Cell Viability Assay

CCK-8 (Dojindo Laboratories; Kumamoto, Japan) was used to evaluate the viability and proliferation of THP-1 cells. THP-1 cells were plated in 96-well microtiter plates at a density of 1 × 10^4^ cells per well with 100 μL of medium containing different concentrations of glucose. After 24, 48, and 72 h of culturing of cells under different concentrations of glucose, 10 μL of CCK-8 solution was added into each well, and the 96-well microtiter plates were incubated for another 4 h. Absorbance was measured at 450 nm using a microplate reader (Bio-Tek instruments, Winooski, VT, USA). The cell survival ratio was expressed as a percentage of the NG control.

### 4.3. ROS Measurement

The levels of ROS were determined using a fluorescent probe, 5-(and-6)-chloromethyl-2′,7′-dichlorodihydrofluorescein diacetate acetylester (CM-H2DCFDA, Invitrogen by Thermo Fisher Scientific, Eugene, OR, USA). THP-1 cells were incubated with 10 µM CM-H2DCFDA for 40 min at 37 °C, then washed thrice with Hanks’ Balanced Salt Solution (HBSS). After washing, cells were resuspended in HBSS at 1 × 10^6^ cells/mL and analyzed by flow cytometry (FACSAria III, BD Biosciences, San Jose, CA, USA). Data were analyzed using the FlowJo software (Tree Star, Inc., Ashland, OR, USA) and represented as a fold change of the mean fluorescence intensity (MFI).

### 4.4. Real-Time Quantitative PCR

Total RNA was extracted using the RNAiso Plus (Takara Bio Inc., Shiga, Japan) reagent. RNA concentrations were measured using the NanoDrop 2000 spectrophotometer (Thermo Fisher Scientific, Wilmington, DE, USA). The cDNA was synthesized with 1 μg RNA using the PrimeScript II 1st strand cDNA kit (Takara). Real-time quantitative PCR was performed using the TB Green premix Ex Taq (Tli RNaseH Plus) (Takara) with a CFX96™ Real-Time System (Bio-Rad Laboratories, California, USA). All gene expression values were normalized to those of glyceraldehyde 3-dehydrogenase (GAPDH). Primer sequences are displayed in Table 1. Details on the PCR analysis are available in the Supplementary Materials. 

### 4.5. Preparation of Protein Samples, Supernatants, and Western Blotting

After an acute glucose shift for 24 h, cells were harvested in ice-cold cell lysis buffer, CelLytic M (Sigma) containing protease and phosphatase inhibitors (Cell Signaling Technology Inc., Boston, MA, USA). Cells were lysed on ice for 30 min and then centrifuged at 14,000× *g* for 30 min at 4 °C. Clear total cell lysates were collected and quantified using the BCA assay kit (Thermo Fisher Scientific, Rockford, IL, USA). For the detection of secreted IL-1β in cell culture supernatants, 5 mM of ATP (Sigma) was added to the cells for the last 30 min, following which the supernatants were collected. The supernatants were concentrated using trichloroacetate (Sigma) or Amicon Ultra-4 (3K MWCO) (Merck Millipore Ltd. Darmstadt, Germany). The total cell lysate (20 μg) and supernatants were loaded onto NuPAGE^TM^ 4–12% bis-tris gradient gel (NOVEX by life technologies, Carlsbad, CA, USA), electrophoresed under reducing conditions, and transferred to nitrocellulose membranes (GE Amersham, MA, USA). Membranes were blocked with 5% BSA (MP Biomedicals, Solon, OH, USA) or 5% nonfat dry milk (LPS solution, Daejeon, Korea) at room temperature for 1 h and incubated with primary antibodies against NLRP3 (AG-20B-0014-C100, 1:5000) (AdipoGen, San Diego, CA, USA), caspase-1 (sc56036, 1:200) (Santa Cruz Biotechnology, Dallas, TX, USA), IL-1β (12242, 1:1000), JNK (9252, 1:1000), p-JNK (4668, 1:1000), NF-κB (4764, 1:1000) p-NF-κB (3033, 1:1000), p38 (8690, 1:1000), p-p38 (4511, 1:1000) (Cell Signaling Technology, Boston, MA, USA), and β-actin (ab8227, 1:10,000) (Abcam, Cambridge, UK) overnight at 4 °C and then incubated with the corresponding horseradish peroxidase-linked secondary antibodies for 1 h at room temperature. The signals were developed using a standard chemiluminescence detection reagent (Thermo Fisher Scientific). We scanned the X-ray film with HP LaserJet Professional M1213nf MFP and quantified the intensities of bands using Image J software (NIH, Bethesda, MD, USA). 

### 4.6. ELISA

The secretion of IL-1β and caspase-1 in supernatants was measured using the ELISA kit (R&D Systems, Inc. Minneapolis, MN, USA), according to the manufacturer’s instructions. 

### 4.7. Statistical Analysis

Data are expressed as the mean ± SEM. Statistical calculations were performed using the GraphPad Prism 7 (GraphPad Software, Inc., San Diego, CA, USA). Data were analyzed by one-way ANOVA followed by Tukey’s multiple comparisons test. *p* < 0.05 was considered statistically significant. * *p* < 0.05, ** *p* < 0.01, *** *p* < 0.001

## Figures and Tables

**Table 1:** Primer sequences for real-time quantitative PCR.

Gene	GenBank Accession No.	Primer sequences (5′-3′)
NLRP3	NM 001127462	F: ACAGCCACCTCACTTCCAG
		R: CCAACCACAATCTCCGAATG
Caspase-1	NM 001257119	F: GCACAAGACCTCTGACAGCA R: TTGGGCAGTTCTTGGTATTC
ASC	NM 145182	F: TGGATGCTCTGTACGGGAAG R: TGGATGCTCTGTACGGGAAG
IL-1β	NM 000567	F: GCCCTAAACAGATGAAGTGCTC R: GAACCAGCATCTTCC CAG
GAPDH	NM 002046	F: CATGAGAAGTATGACAACAGC R: AGTCCTTCCACGATACCAAAGT

## Data Availability

The data presented in this study are available in the manuscript or supplementary material.

## Supplementary Materials

The following are available online at https://www.mdpi.com/article/10.3390/ijms22189952/s1, Materials and method, Figure S1: Acute glucose shift induces the activation of the NLRP3 inflammasome in THP-1 cells, Figure S2: Hyperosmolarity did not affect the activation of the NLRP3 inflammasome, Figure S3: The effect of acute glucose shift on ERK1/2 phosphorylation in THP-1 cells.
