# Supplementary material for: Acute Glucose Shift Induces the Activation of the NLRP3 Inflammasome in THP-1 Cells
Source: Int J Mol Sci. 2021 Sep 15;22(18):9952. doi: 10.3390/ijms22189952 (PMC8465199; doi:10.3390/ijms22189952)
Supplement: Supplementary file 1 [file ijms-22-09952-s001.zip › ijms-1387795-supplementary.pdf]

Supplementary Methods

Real-time Quantitative PCR Method

RNA concentrations and the 260/280 ratio used as the purity indicator for RNA were measured using the NanoDrop 2000 spectrophotometer.. For total RNA extraction, we added the RNAiso Plus reagent to cells and frozefreezed the cells in a deep freezer . The cells. Cells were then thawed and chloroform was added to the cells. After mixing well, the samplessample were incubated at room temperature and then centrifuged . The aqueous phase of the sample was withdrawntaken and one volume of 70% ethanol was added. The mix-turemix of the aqueous phase and ethanol were loaded onto theto RNeasy column manufactured byfrom Qiagen GmbH (Hilden, Germany).

A real-time qPCR was performed using the following program:

1: 95℃ for 3:00

2: 95℃ for 0:10

3: 60℃ for 0:30

4. 72℃ for 0:30

5: Go to step 2, 35 cycles amplification

6: 95℃ for 0:10

7: Melt curve 65℃ to 95℃; Increment: 0.5℃ for 0:05

THP-1 Cell Differentiation Method

THP-1 cells were differentiated with 5 ng/ml phorbol-12-myristate 13-acetate (PMA, Sig-ma-Aldrich, Saint Luis, MO, USA) for 72 h followed by 48 h incubation in RPMI medium and then exposed to acute glucose shift for 24 h.

Method to Isolate Peritoneal Murine Macrophages

Male C57BL/6 mice (4 weeks old) were purchased from Orient (Seongnam, Korea) and acclimated under controlled conditions for 2 weeks before the experiment. All mice between 6-8 weeks of age were utilized. The animals were housed in cages located in temperature-controlled rooms under 12:12 h light–dark cycle conditions and fed water and food ad libitum. All animal procedures were approved by the Ajou University Animal Care Ethics Committee (Ethics No. 2019-0013). Macrophages were isolated from the peritoneal exudate cells and cultured as described pre-viously (Nutrients 2017, 9, 1370; doi:10.3390/nu9121370).

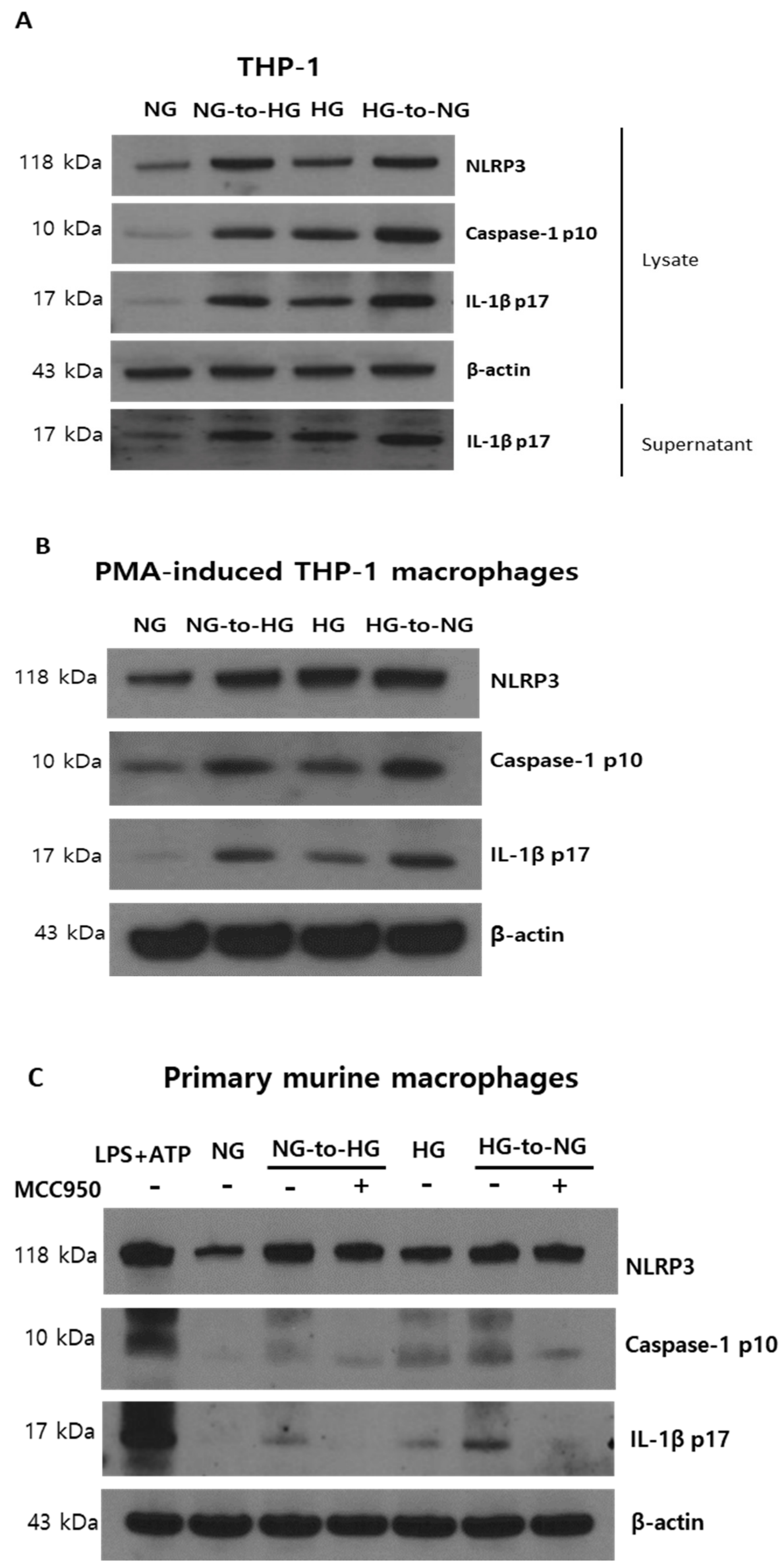

Figure S1. (A) Acute glucose shift induces the activation of the NLRP3 inflammasome in THP-1 cells. The media of the THP-1 cells initially designated as NG- and HG-cultured cells were changed to HG

and NG media, respectively. The cell lysates and supernatants were subjected to western blotting using antibodies against NLRP3, caspase-1 p10, and IL-1β p17. (B) THP-1 cells were differentiated with 5 ng/ml phorbol-12-myristate 13-acetate (PMA, Sigma-Aldrich, Saint Luis, MO, USA) for 72 h followed by 48 h incubation in RPMI medium and then exposed to acute glucose shift for 24 h. The cell lysates were subjected to western blotting using antibodies against NLRP3, caspase-1, and IL-1β. (C) Primary murine macrophages were pretreated with MCC950 (10 μM) for 1 h and then exposed to glucose shift for 24 h. LPS-treated peritoneal macrophages were incubated with ATP (5 mM) for 30 min as positive control. The cell lysates were subjected to western blotting using antibodies against NLRP3, caspase-1, and and IL-1β.

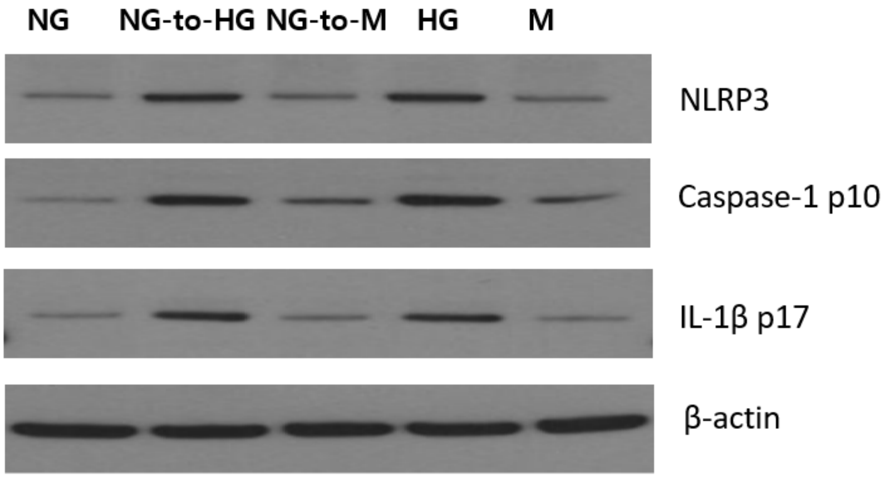

Figure S2. Hyperosmolarity did not affect the activation of the NLRP3 inflammasome. To determine whether increased osmolality affects the activation of the NLRP3 inflammasome, cells were exposed to glucose shift, high glucose, or mannitol for 24 h. The cells were grouped as follows: NG (5.5 mM of glucose), NG-to-HG (5.5 mM to 25 mM of glucose), NG-to-M (cells cultured in media containing 5.5 mM of glucose and then grown in media containing 5.5 mM glucose + 19.5 mM mannitol), HG (25 mM of glucose), or mannitol (25 mM of mannitol). After 24 h, the protein expression of NLRP3, caspase-1 p10, and IL-1β p17 were detected using western blotting.

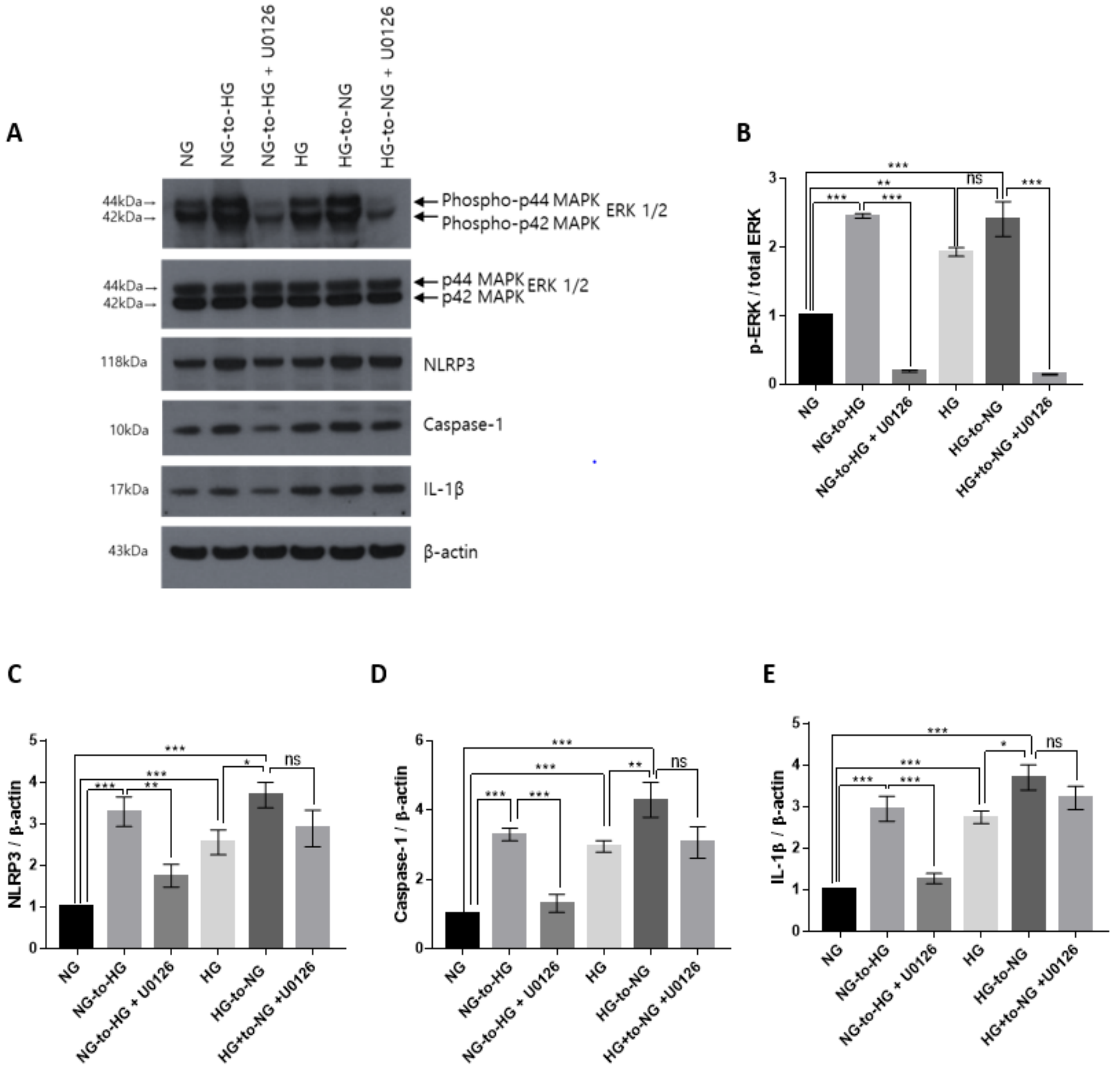

Figure S3. The effect of acute glucose shift on ERK1/2 phosphorylation in THP-1 cells. THP-1 cells were pretreated with the ERK1/2 inhibitor, U0126 (10 μM), for 2 h and then exposed to acute glucose shift for 24 h. (A) The cell lysates were subjected to western blotting using antibodies against p-ERK1/2, NLRP3, caspase-1, and IL-1β. (B-E) The protein expression levels of p-ERK, NLRP3, caspase-1, and IL-1β were quantified using Image J. Data are presented as the mean ± SEM from at least three independent experiments. * $P < 0.05$, ** $P < 0.01$, *** $P < 0.001$, ns, not significant.
